# The Mechanism of Ochratoxin Contamination of Artificially Inoculated Licorice Roots

**DOI:** 10.3390/toxins15030219

**Published:** 2023-03-13

**Authors:** Abdelrahman Elamin, Hirofumi Enomoto, Maiko Watanabe, Shohei Sakuda

**Affiliations:** 1 Department of Biosciences, Teikyo University, 1-1 Toyosatodai, Utsunomiya 320-8551, Japan; 2 Advanced Instrumental Analysis Center, Teikyo University, 1-1 Toyosatodai, Utsunomiya 320-8551, Japan; 3 Division of Microbiology, National Institute of Health Sciences, Kanagawa 210-9501, Japan

**Keywords:** ochratoxins, licorice root, DESI-MS/MSI, HPLC, SEM

## Abstract

Ochratoxin (OT) contamination of medicinal herbs is a serious threat to human health. This study was performed to investigate the mechanism of OT contamination of licorice (*Glycyrrhiza* sp.) root. Licorice root samples were cut into eight parts, which were placed separately on sucrose-free Czapek Dox agar medium, inoculated with the spores of ochratoxigenic *Aspergillus westerdijkiae*. After incubation for 10 and 20 days, the OT contents of the samples were determined by high-performance liquid chromatography, and microtome sections prepared from the samples were analyzed by desorption electrospray ionization tandem mass spectrometry, to visualize OT localization. The same sections were further examined by light microscopy and scanning electron microscopy, to investigate the path of fungal mycelial penetration of the inner roots. OT concentrations tended to increase from the upper- to the middle-root parts. OTs were located in cut areas and areas of cork layer damage; they were not present in the undamaged cork layer, indicating that the structure of this layer prevents OT contamination of the licorice root.

## 1. Introduction

Medicinal herbs are used worldwide. In developing countries, they serve as primary healthcare resources; while in developed countries, they tend to be used for self-medication [1]. Consequently, prevention of medicinal herb contamination by harmful substances such as mycotoxins is of concern [2,3]. Licorice is an herbaceous perennial that has been used since ancient times as a medicinal remedy. As part of the family Fabaceae, it is categorized into three *Glycyrrhiza* species: *Glycyrrhiza uralensis* Fisch., *Glycyrrhiza inflata* Bat., and *Glycyrrhiza glabra* L. [4]. The most important medicinal parts of licorice are its rhizomes and roots. When applied alone, or in combination with other herbs, licorice has been used to treat gastric ulcers, sore throat, cough, bronchitis, and arthritis [5,6]. Natural medicines prepared from *Glycyrrhiza* sp. roots are also important components of many traditional Chinese (Kampo) medicine preparations [7]. However, licorice root is easily contaminated by fungus-producing mycotoxins, due to its direct contact with soil [3,8].

Mycotoxins, one of the most serious contaminants of natural origin, are produced by toxigenic fungal strains, mostly *Aspergillus*, *Penicillium*, and *Fusarium* [9]. The mycotoxins that are most damaging to medicinal herbs are aflatoxins, ochratoxins (OTs), fumonisins, zearalenone, and deoxynivalenol [2]. The recent detection of ochratoxin A (OTA) in licorice is currently considered the most relevant contaminant [10]. There are various factors which facilitate the production of OTs by fungi present in licorice roots. These include: extended drying times, elevated moisture, poor production techniques and storage facilities, as well as failure to use antifungal pesticides during planting. Production of OTs can lead to serious adverse economic and health effects [11].

OTA is a toxic secondary metabolite that is produced by several strains of fungi. Notably, by *Penicillium verrucosum* in temperate climates, and by *Aspergillus ochraceus*, *Aspergillus carbonarius*, and *Aspergillus niger* in tropical regions [12]. The optimum conditions for enhanced OT production are, pH 3–10, a temperature of 31 °C, and a minimum of 0.8 water activity. Meanwhile, production of OTs by *P. verrucosum* is optimal at pH 6–7, a temperature of 20 °C, and a minimum of 0.86 water activity. OT production is also enhanced in the presence of iron, zinc, and copper [13]. Ochratoxigenic strains of the *Penicillium* and *Aspergillus* genera have been isolated from dry licorice root samples [14].

Among the OT family, OTA is a naturally abundant and harmful fungal metabolite. It is also a common contaminant of several medicinal herbs. Concentrations of OTA in food have been analyzed over the past 10 years in 29 European countries. Based on a total of 71,769 analyses, higher concentrations of OTA have been found in plant extract formula flavorings or essences which contain licorice extracts [15]. Currently, maximum OTA levels in licorice are strongly regulated in the European Union. For example, Commission Regulation (EU) No. 105/2010, amending Regulation 1881/2006, sets the maximum levels of OTA in licorice root, and licorice extracts used in licorice confectionery, at 20 µg/kg and 80 µg/kg, respectively [10,16]. When *Glycyrrhiza* sp. root samples, and sweets containing licorice extract, were analyzed in Germany, more than 50% contained OTA at concentrations ranging from 0.3–216.0 µg/kg [17]. Furthermore, among licorice root and derived products analyzed in Spain (*n* = 30), all contained OTA, with concentrations of up to 252.8 µg/kg detected [18].

Ideally, analytical methodologies for quantification of OTs should be rapid, selective, simple, accurate, and sensitive. To date, liquid chromatography methods, employing different detectors [e.g., mass spectrometry (MS), diode array detector (DAD), and fluorescence detection (FLD)], have been key for OT analysis. To quantify OTs, gas chromatography (GC) is utilized, albeit to a lesser extent. To perform screening of OTs in food samples, GC with flame ionization detection (GC-FID) and GC with tandem MS (GC-MS/MS) have been used [19]. For cleanup and isolation of OTs extracted from foods and biological fluids, immunoaffinity columns (IACs) are widely used. These columns are prepared by binding antibodies which recognize a particular OT, to a specially activated solid-phase support. Additionally, a support suspended in an aqueous buffer solution is packed into a cartridge. OTs present in an extract or fluid that bind to the immobilized antibodies are retained, while impurities are removed, with water or an aqueous solution. Bound OTs can subsequently be displaced and collected, when a miscible solvent such as methanol is added to the column [20].

To prevent contamination of licorice roots, the mechanism(s) mediating OT contamination need to be elucidated. The goals of the current study were to examine: (1) the susceptibility of different parts of the licorice root to OT contamination, (2) the localization of OTs in contaminated licorice roots, and (3) the path of fungal mycelial penetration of licorice roots. To detect OTs in different regions of the licorice root, HPLC was performed. OT distribution among different sections of the licorice plant was further determined using desorption electrospray ionization tandem MS (DESI-MS/MS). Once accumulation of OTs in the sections of the tested samples was confirmed, the same sections were examined with scanning electron microscopy (SEM) and light microscopy (LM), to determine the paths by which fungi had entered the samples, as well as the relationship between fungal mycelia accumulation and OT production.

## 2. Results

### 2.1. Susceptibility of Different Licorice Root Parts to OT Contamination

Licorice root samples were artificially contaminated with OTs, using a previously reported method [21]. Four licorice roots of similar size and shape were washed with ethanol:water (1:1, *v*/*v*) soon after harvest. Each root was divided into eight 5 cm pieces, which were halved (creating 2.5 cm pieces) and desiccated in an oven at 50 °C. The pieces were then placed separately on sucrose-free Czapek Dox agar (CZA) medium, inoculated with the spores of ochratoxigenic *Aspergillus westerdijkiae* strain NIHS-7241 (Figure S1). This strain was isolated from soil attached to licorice roots harvested in Tumxuk, Xinjiang, China. After incubation at 25 °C, for 10 and 20 days (Figure S1), fungal growth on the root samples was observed. The fungal mycelia accumulated in the cut areas of each root piece. After washing, desiccation, and grinding, OTs in the samples were quantified by high-performance liquid chromatography (HPLC).

**Figure 1:**
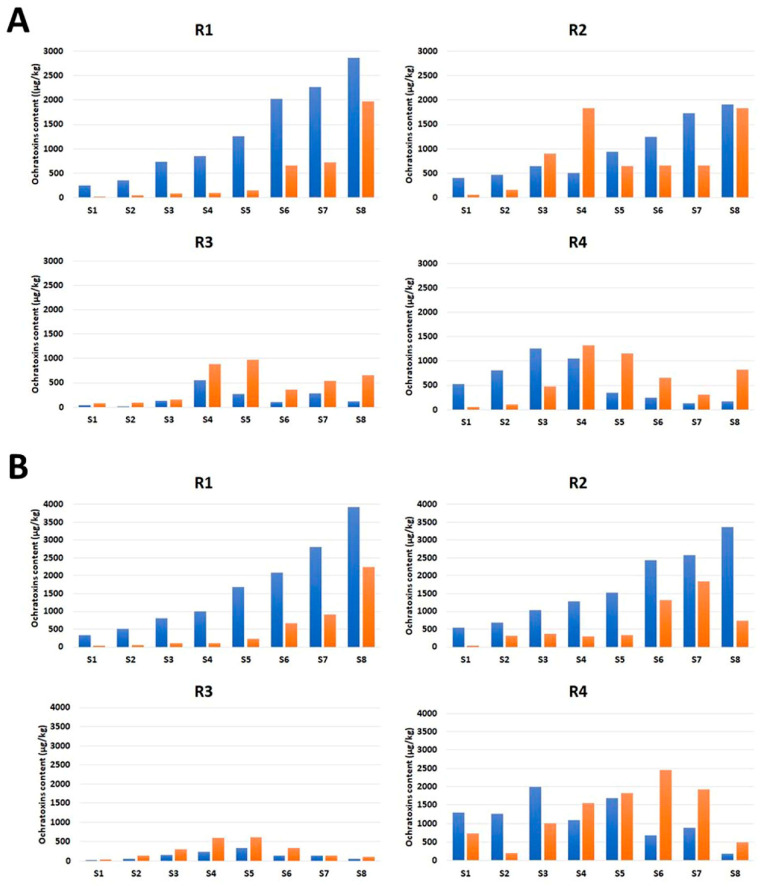
Ochratoxin A (blue) and B (orange) contents in licorice root (R1–R4) samples (S1–S8 from top to bottom), after 10 (**A**) and 20 (**B**) days of incubation.

The OT contamination tendencies differed between the upper (S1–S4) and lower (S5–S8) halves of the four tested roots (R1–R4), after 10 and 20 days of incubation (Figure 1). In the upper root halves, OT concentrations increased gradually in the top–middle direction in all tested roots. In the lower halves, two roots (R1 and R2) showed the same tendency, whereas the other two roots (R3 and R4) had fluctuating OT concentrations along their lengths. This fluctuation was accompanied by differences in the ochratoxin B (OTB):OTA ratios from those for the upper halves of the same roots and the lower halves of R1 and R2.

### 2.2. OT Localization in Contaminated Licorice Roots

**Figure 2:**
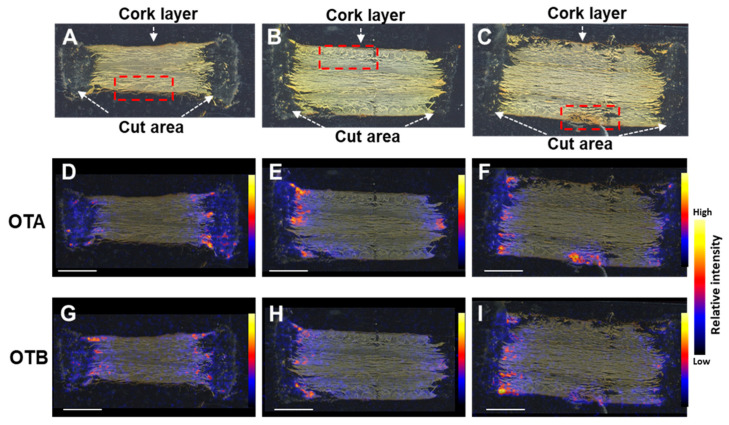
Visualization of OTA and OTB in contaminated licorice root sections S2 (**A**,**D**,**G**), S3 (**B**,**E**,**H**), and S5 (**C**,**F**,**I**). (**A**–**C**) Optical images. Desorption lectrospray ionization tandem mass spectrometry images of OTA (*m/z* 402.07 → 358.08) (**D**–**F**) and OTB (*m/z* 368.11 → 324.2) (**G**–**I**). Dashed red squares in (**A**–**C**) indicate the areas magnified in Figure 3A–C; cork layer damage can be seen in the square in (**C**). Scale bar = 5 mm.

The distribution of OTs within contaminated crude material is of high interest, to obtain insight into infection mechanisms and the possibility of reducing contamination during its processing [22]. To investigate the localization of OTA and OTB in the contaminated licorice roots, DESI-MS/MS was performed. In the MS/MS spectra of authentic OTA and OTB samples, relative intensities of fragment ion peaks at *m/z* 358.0818 and 324.1210, respectively, corresponding to [M–COOH]^−^ ions, were highest (Figure S2). A licorice root was cut into eight parts and inoculated with *A. westerdijkiae,* using the method described in Section 2.1. After 10 days of incubation, sections, cut along the direction of the root to involve the cork layers on both sides, were prepared from three root parts (S2, S3, and S5; Figure 2A,D,G and Figure 2B,E,H, and Figure 2C,F,I, respectively). Fragment ions corresponding to [M–COOH]^–^ ions, for OTA and OTB, were detected in the MS/MS spectra of these sections (Figure S3). The ion images showed little difference between the OTA and OTB localization patterns. The OTs were localized predominantly around the cut areas, in damaged parts of the cork layer, and extending slightly into it (Figure 2F,I). These results suggest that the cork layer protected against OT contamination of the inner parts of the roots. HPLC analysis of the remaining parts of S2, S3, and S5, showed that the samples’ OTA contents were 942, 983, and 1436 μg/kg, respectively.

### 2.3. Path of Fungal Mycelial Penetration of Licorice Root

**Figure 3:**
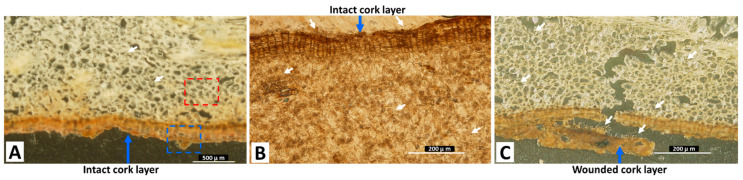
Light microscopic images of areas demarcated by dashed red square areas in Figure 2A (**A**), Figure 2B (**B**), and Figure 2C (**C**).

The sections visualized by DESI-MS/MS were further analyzed by light microscopy (LM) and scanning electron microscopy (SEM), to investigate the path of fungal mycelial penetration to the inner parts of the roots. Figure 3A shows the LM image of the dashed red square area in Figure 2A, showing the accumulation of the fungal mycelia in inner root cells. The LM images of the dashed red square areas in Figure 2B,C, also show the fungal mycelial accumulation in inner root cells. The fungal mycelia accumulated at the outer layer of the cork and penetrated the wounded cork layer, as observed in Figure 3B,C, respectively.

**Figure 4:**
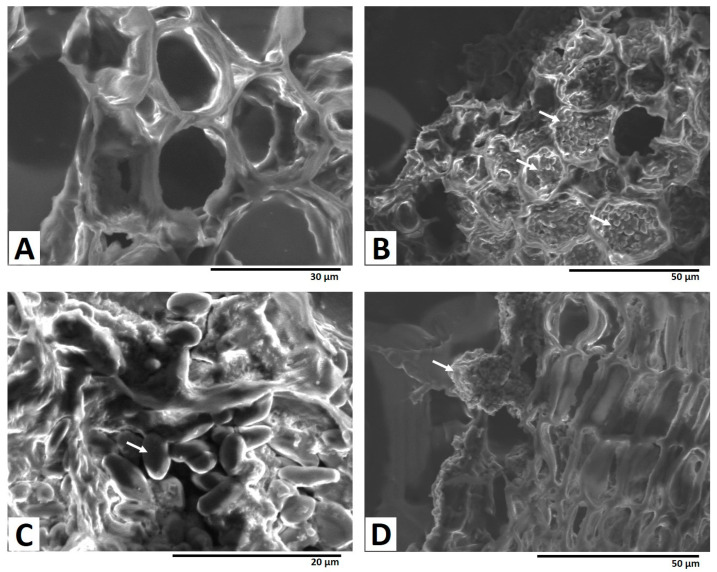
SEM images of fungal mycelial penetration of licorice root. (**A**) Control without fungal inoculation. (**B**,**C**) Area demarcated by the dashed red square in Figure 3A. (**D**) Area demarcated by the dashed blue square in Figure 3A. White arrows show fungal spores.

Figure 4B,C show the SEM images of the dashed red square in Figure 3A, clearly showing the accumulation of the fungal spores in inner root cells. Fungal spores were also observed at the outer layer of the cork in the SEM image (Figure 4D), and no penetration occurred to the inner part of the section from the cork layer. These observations suggest that the fungal mycelia penetrated to the depths of the licorice sample, in contrast to the pattern of OT accumulation.

White arrows show fungal mycelial accumulation in cells. The dashed red and blue squares in (A) indicate the areas shown in Figure 4B–D, respectively.

## 3. Discussion

*A. westerdijkiae* is one of the most common producers of OTs, which contaminate fresh and dry licorice roots that are cultivated in Xinjiang, China [14]. In most of the licorice roots we tested, *A. westerdijkiae* production of OTA was present at a higher level than OTB. However, in some parts of the roots, production of OTB was higher than OTA. Clarification of the ratio of OTA to OTB that is produced by *ochratoxigenic fungi* in certain fungal media and natural materials, is of interest to relevant researchers. In artificial and natural media, *A. ochraceus* and its taxon (*A. westerdijkiae*) produce less OTB than OTA, with ratios ranging from 1:2 to 1:34 [23]. Under specific culture conditions, such as high sucrose (32%) in 2% yeast extract solution, levels of OTB and OTA production are comparable [24]. However, higher OTA than OTB concentrations have been detected in products such as cereals, cacao, and coffee [25]. In a few cases, such as in 11 of 20 samples of red wine from Spain, the OTB production level was found to be higher than that of OTA [26].

DESI-MS/MS analysis of licorice samples in the present study succeeded in precisely determining the distribution of OTs in their sections. Localization of OTs in contaminated materials has been investigated in previous studies with different tools. For example, an OTA analysis of parts of naturally infected sausages, by LC-MS/MS, showed that OTA was only present in the casings, and did not reach the stuffed meat [27]. When French semi-hard Comté cheese was artificially inoculated with *P. verrucosum* and analyzed by Q-TOF/LC/MS, the maximum OTA concentration detected was near the surface of the cheese. Only slight OTA production was detected at greater depths of the cheese, after a longer incubation period [28]. More recently, mass spectrometry imaging (MSI) has been used to determine the spatial distribution of mycotoxins in tissues from plants [29]. A matrix-assisted laser desorption/ionization (MALDI)-MSI analysis of OTA contamination in various vegetable foodstuffs, demonstrated that OTA co-localized with visible fungal spoilage [22]. These results are consistent with those of the current study, which demonstrated that OTs can accumulate in the cut parts and wounds of samples. The DESI-MS/MS analysis performed in the present study, further demonstrated that OTs were only slightly concentrated at greater depths in the samples examined, despite clear fungal penetration being shown by microscopic examination.

The process of fungal penetration into plant roots is complex, primarily because fungal invasion mechanisms are countered by strong defense mechanisms. Roots develop both physical and chemical barriers to block pathogen progression, especially at the cork layer. Roots produce antifungal compounds (e.g., glabridin in licorice), they mediate the conversion of cell walls into cork tissue with expression of suberin (suberization), cell walls undergo lignification, and gum accumulates [30,31]. Correspondingly, the healthy cork layer of licorice roots exhibits resistance to infection by *A. westerdijkiae*, and fungus was unable to penetrate it throughout the incubation period. In contrast, fungal pathogens have developed diverse invasion mechanisms to penetrate various structures of roots. Fungal pathogens can attack cuts, wounds, and stomata of roots, to seek sources of nutrients for growth and development [32]. By forming infection structures, called hyphal differentiations, fugal pathogens can penetrate different types of cell walls. Briefly, accumulation of hyphae can form an infection pad, which forms just prior to penetration of hyphae. The latter involves minor modifications of their morphology. The resulting increase in pressure within the infection structure further supports the penetration process [33]. A previously described mechanism for fungal penetration of plant roots is consistent with the observations of the present study regarding contaminated licorice samples. *A. westerdijkiae* infected the cut parts and wounds of the licorice samples examined. Fungal mycelia subsequently accumulated in the infected areas, with infection cushions formed according to visual assessment. After the fungal hyphae penetrated the inner parts of the samples, spores formed in the licorice inner cells, and were observed with LM and SEM.

**Figure 5:**
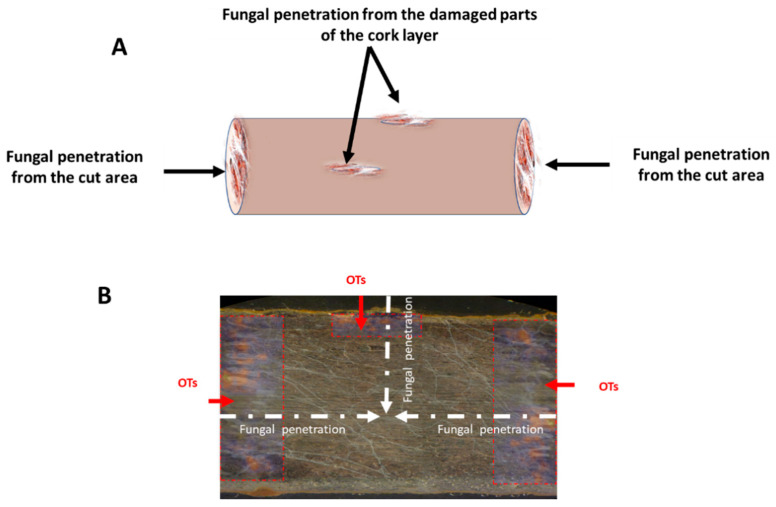
Putative mechanism of fungal and OT contamination of licorice root. (**A**) Direction of fungal penetration. (**B**) Thin section of licorice root showing fungal and OT contamination.

Figure 5 presents a putative mechanism of fungal and OT contamination of licorice root according to the findings of the present study. DESI-MS/MS detected high concentrations of OTs in cut root areas and in damaged areas of the cork layer. Meanwhile, lower levels of OTs were detected in the inner root and cork layer. Taken together, these results suggest that the former represent the main areas attacked by the fungus and they were highly contaminated with OTs. Meanwhile, the structure and high lignin content of the cork layer of licorice root was not easily penetrated by fungal mycelia [34]. However, LM and SEM images did show that fungal mycelia had almost completely penetrated the root after 10 days of incubation. During this process, cell walls of the root had undergone collapse or destruction. These results suggest that areas of OT accumulation and fungal growth do not coincide.

## 4. Conclusions

The results of the present study demonstrate a mechanism of OT contamination of artificially inoculated licorice roots. Chromatography analysis confirmed varying susceptibilities of the different parts of the licorice root to contamination with OTs, according to their location in the root. DESI-MS/MS also showed that OTs localized to cut areas and areas of cork layer damage, suggesting that the cork layer of the licorice root provides protection against OT contamination. However, despite fungal penetration into the samples, that was clearly observed by microscopic study, OTs were found to accumulate near the surface of the samples, and this accompanied visible mycelial growth.

## 5. Materials and Methods

### 5.1. OT Productivity of A. Westerdijkiae Strain NIHS-7241 Isolated from Licorice

One of the isolates derived from licorice root harvested in Tumxuk, Xinjiang, China was identified by molecular and morphological methods (Supplementary Materials) as *A. westerdijkiae,* and used as the strain NIHS-7241. The OTA and OTB productivity of the NIHS-7241 strain was tested by inoculating 2 mL liquid medium (potato dextrose broth (PDB) and malt peptone broth (MPB), 17 g/L malt extract, and 3 g/L Bacto^TM^ peptone; Difco^TM^) in a 12-well microplate, with 2 µL *A. westerdijkiae* NIHS-7241 spore suspension (10^7^/mL). The microplates were incubated at 25 °C for 7 days. After incubation, the culture broth from each well was filtered through glass-fiber filter paper (GA-100) into a 10 mL Labcon centrifuge tube. Hydrochloric acid (0.1 M, 1 mL; Kanto Chemical Co., Inc., Tokyo, Japan) was added to the filtrated solution and mixed, followed by the addition of 1 mL chloroform (Kanto Chemical Co., Inc., Tokyo, Japan). The mixture was vortexed and left at room temperature for 30 min to split into two layers. Chloroform solution (500 µL) was transferred from the lower layer into an Eppendorf tube and evaporated. The residue was dissolved in 400 µL water:acetonitrile:acetic acid solution (51:48:1, *v*/*v*/*v*), and the solution was analyzed by HPLC using the parameters described in Section 5.3. The OTA productivity values for this strain, in the liquid media (PDB and MPB), were 0.2 and 0.1 μg/kg, respectively. OTB production by the strain was not detected under the analytical conditions.

### 5.2. Sample Preparation

Six roots of similar shape and length (40 cm), selected from licorice roots cultivated for 17 months in Ibaraki, Japan, were used in the experiments. Two roots were used for the control and spiked experiment, and four roots were used for the experiment described in Section 2.1. Each of these four roots was divided into eight 5 cm pieces, which were labeled sequentially from top to bottom (S1–S8), and each piece was divided into two 2.5 cm pieces, for use in two incubation periods. The root samples were soaked in ethanol:water solution (1:1, *v*/*v*) for 30 min and desiccated in an oven at 50 °C. They were then placed on sucrose-free CZA medium in Petri dishes (150 mm × 25 mm) and spread with 250 µL *A. westerdijkiae* NIHS-7241 spore suspension (10^7^/mL). The Petri dishes were incubated at 25 °C for 10 and 20 days. After incubation, the samples were washed with ethanol:water solution (1:1 *v*/*v*) and desiccated in an oven at 50 °C, followed by HPLC analysis. In the experiment in Section 2.2 and Section 2.3, one root was cut into eight pieces, washed with ethanol:water (1:1, *v*/*v*), desiccated in an oven at 50 °C, and incubated using the method described in Section 2.1. After 10 days of incubation at 25 °C, three pieces of the root (S2, S3, and S5) were washed with ethanol:water (1:1, *v*/*v*), desiccated in an oven at 50 °C, and examined by DESI-MS/MS, LM, and SEM.

### 5.3. OT Quantification by HPLC

The root samples were ground (Wonder Crusher WC-3; Osaka Chemical Co., Ltd., Osaka, Japan), weighed, and mixed with 10 mL of a solution of sodium hydrogen carbonate in water (0.13 M): methanol (50:50, *v*/*v*). The mixture was vortexed for 5 min at room temperature. After centrifugation (4770× *g*, 10 min, 4 °C), phosphate-buffered saline (PBS), containing 0.01% Tween 20 (Kanto Chemical Co., Inc., Tokyo, Japan), was added to 2 mL of the supernatant, to a final volume of 10 mL. The mixture was filtered with glass-fiber filter paper (GA-100; Advantec Toyo Kaisha, Ltd., Tokyo, Japan) and transferred to IAC (Ochraking; Horiba, Ltd., Kyoto, Japan). The column was washed twice with 3 mL PBS and 3 mL water, and eluted with 3 mL methanol:acetic acid solution (98:2, *v*/*v*). The eluate was dried under N_2_ gas and dissolved in 0.4 mL water:acetonitrile:acetic acid solution (51:48:1, *v*/*v*/*v*). The solution was filtered through a 0.2 µm syringe filter (Minisart^®®^ RC 4; Sartorius Stedim Lab. Ltd., Stonehouse, UK) and subjected to HPLC (Capcell Pak C_18_ UG 120 column, 250 × 4.6 mm inner diameter; Osaka Soda Co., Ltd., Osaka, Japan). An isocratic elution of water:acetonitrile:acetic acid (60:40:1, *v*/*v*/*v*), at a flow rate of 1.0 mL, was used for the detection of fluorescence at 318 nm excitation and 460 nm emission from 0.1 to 14 min, and at 333 nm excitation and 460 nm emission from 14 to 22 min. The retention times and limits of detection for OTA and OTB were 10.49 and 15.76 min, and 0.1 and 0.9 µg/kg, respectively.

### 5.4. Thin Section Preparation

Licorice root samples (S2, S3, and S5) were washed with ethanol:water (1:1, *v*/*v*), dipped in a 3% carboxy methyl-cellulose solution, and frozen with liquid nitrogen. Adhesive film (cryofilm type 2C (9); SECTION-LAB Co., Ltd., Hiroshima, Japan) was attached to the frozen sample cross sections, followed by slicing (20 µm thickness) using a cryostat (CM1860; Leica Microsystems, Wetzlar, Germany). The film was then attached to a glass slide (Superfrost Plus; Fisherbrand) with adhesive tape and stored at −80 °C until analysis [35]. The sections were examined by DESI-MS/MS, LM, and SEM.

### 5.5. DESI-MS/MSI

A Synapt XS HDMS Q-TOF mass spectrometer, equipped with a two-dimensional DESI ion source (Waters, Milford, MA, USA), was used for DESI-MS/MSI [36]. DESI solvent (95% methanol containing 10 mM acetic acid) and the following DESI ion source conditions were used: capillary voltage, 3.0 kV; spray impact angle, 80°; spatial resolution, 300 µm; and scanning speed, 200 µm s^−1^. Signals, at *m/z* 100–500, were measured in the negative ion and sensitivity modes. The trap and transfer collision energies were optimized using the standards of 20 and 2 V, for OTA and OTB, respectively. The MS/MS window was maintained approximately at the precursor ion *m/z* ± 2 Da. For section analysis, the target-enhanced mode, at *m/z* 358 and 324, was used, to enhance the detection intensities of the fragment ions. Chemical formulae and fragment ion structures were calculated using the elemental composition and MassFragment functions, respectively, with the MassLynx software (version 4.2; Waters). Ion images were reconstructed using the HDImaging software (version 1.5; Waters).

### 5.6. Light Microscope (LM) and Scanning Electron Microscopy (SEM)

Fungal growth was observed under an Olympus (Tokyo, Japan) SZH stereomicroscope and Hitachi (Tokyo, Japan) TM3030 tabletop microscope (Hitachi, Ltd., Tokyo, Japan).

## Data Availability

The data will be available for request.

## Supplementary Materials

The following supporting information can be downloaded at: https://www.mdpi.com/article/10.3390/toxins15030219/s1, Figure S1: Artificial contamination of four licorice roots for 10- and 20-day incubations at 25 °C. Figure S2: MS/MS spectra of ochratoxin A (**A**) and ochratoxin B (**B**) standards, obtained by desorption electrospray ionization tandem mass spectrometry imaging (DESI-MSI). Figure S3: MS/MS spectra of contaminated licorice root obtained by desorption electrospray ionization mass spectrometry imaging (DESI-MSI). The identification of *Aspergillus* strains isolated from licorice. References [37,38,39,40] are cited in the Supplementary Materials.
