# Supplementary material for: The Mechanism of Ochratoxin Contamination of Artificially Inoculated Licorice Roots
Source: Toxins (Basel). 2023 Mar 13;15(3):219. doi: 10.3390/toxins15030219 (PMC10058647; doi:10.3390/toxins15030219)
Supplement: Supplementary file 1 [file toxins-15-00219-s001.zip › toxins-2193683-supplementary.pdf]

toxins

MDPI

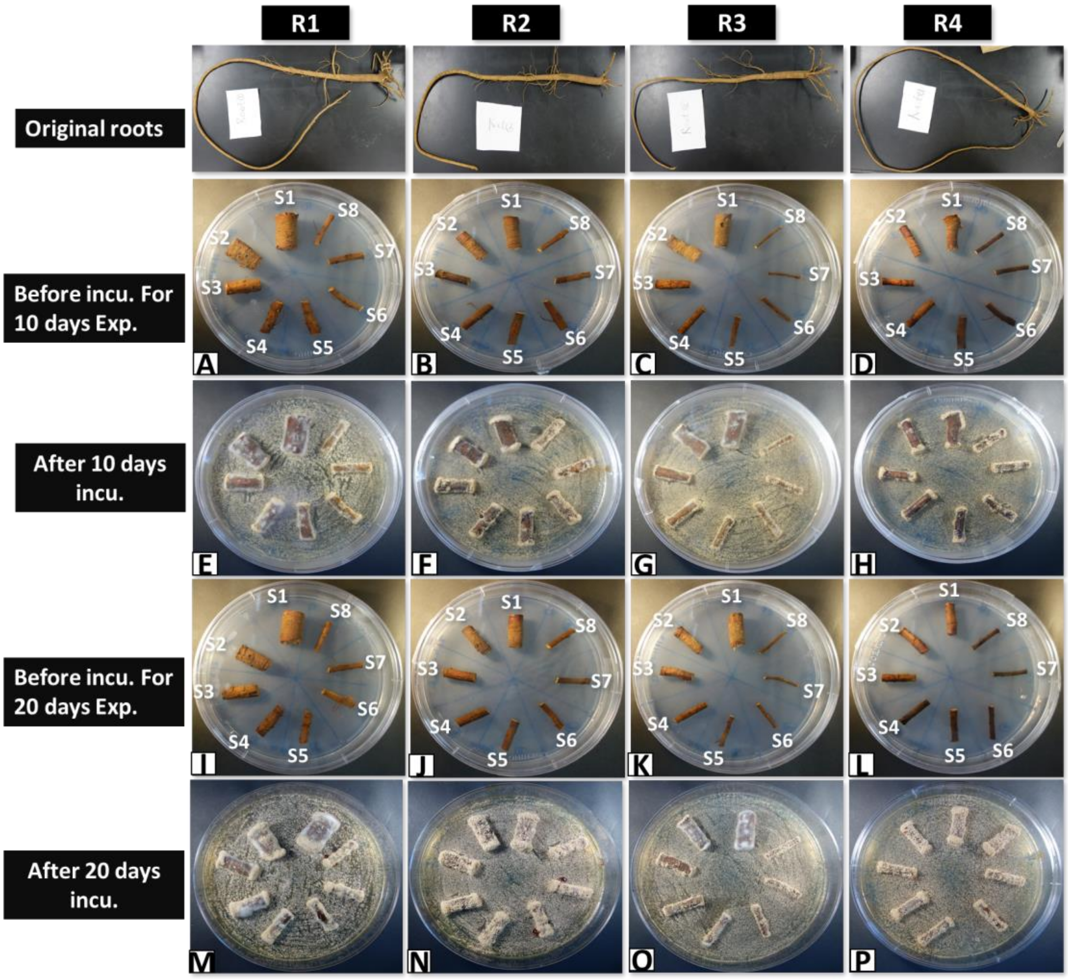

Figure S1. Artificial contamination of four licorice roots for10 and 20-days incubation at 25 °C. (**A**–**D**) the pieces of the four roots on the modified CZA medium inoculated with *Aspergillus westerdijkiae* before 10-days incubation; (**E**–**H**) the pieces of the roots after 10-days incubation; (**I**–**L**) the pieces of the four roots on the modified CZA medium inoculated with *A. westerdijkiae* before 20-days incubation; (**M**–**P**) the pieces of the roots after 20-days incubation.

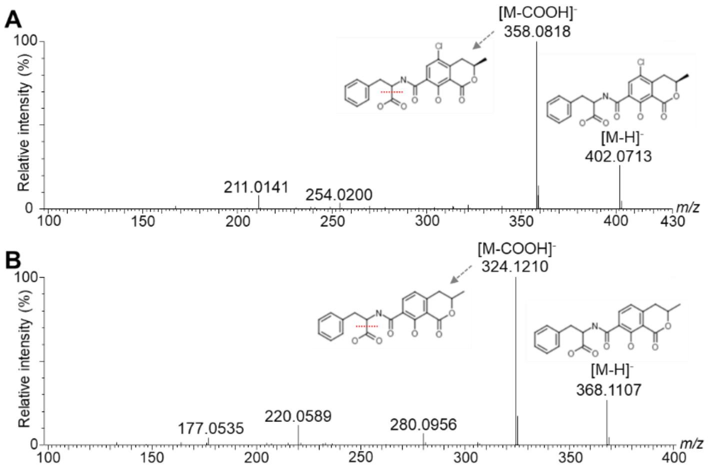

Figure S2. MS/MS spectra of ochratoxin A (**A**) and ochratoxin B (**B**) standards obtained by desorption electrospray ionization - tandem mass spectrometry imaging (DESI-MSI). The collision energy was 20 V.

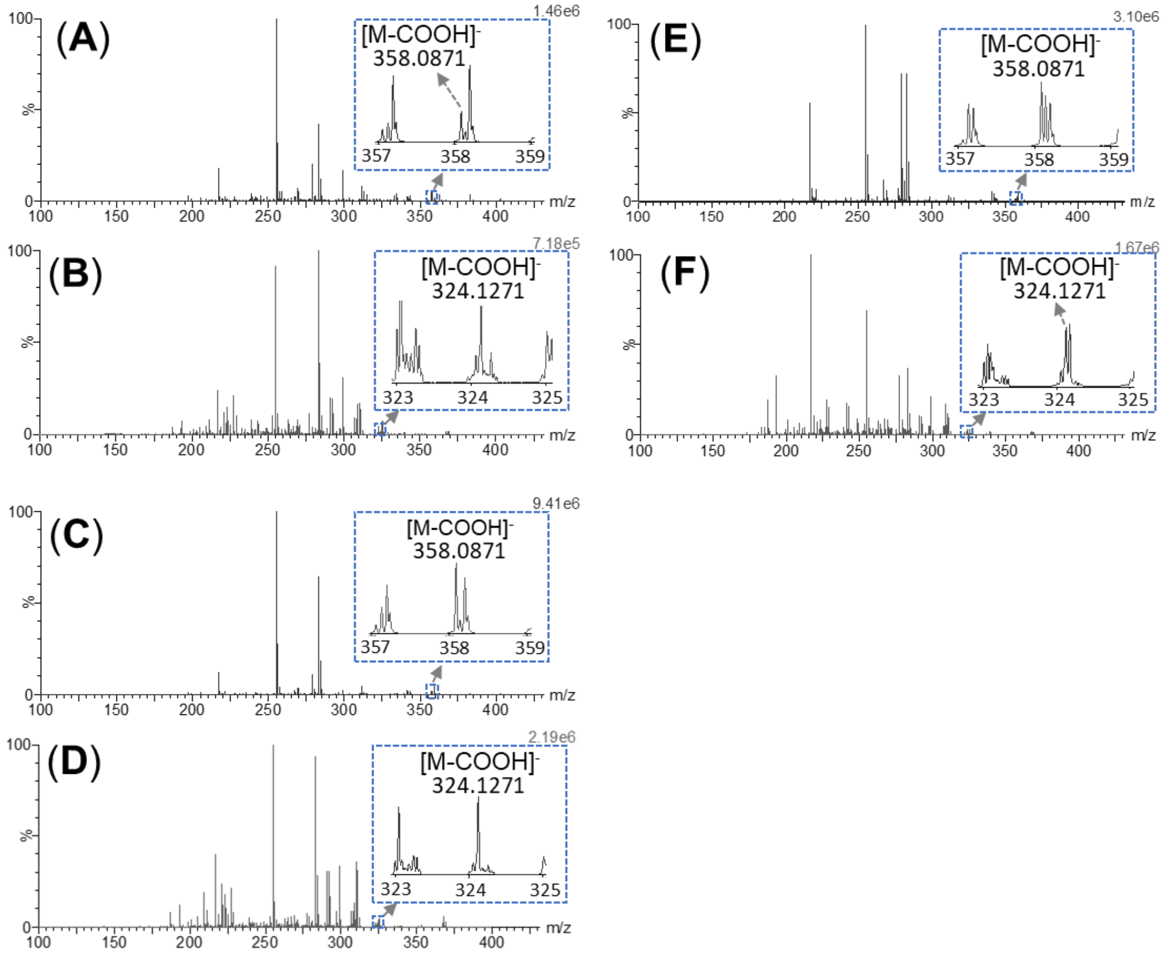

Figure S3. MS/MS spectra of contaminated licorice root obtained by desorption electrospray ionization-mass spectrometry imaging (DESI-MSI). Target-enhanced mode was used. MS/MS spectra of ochratoxin A obtained from S2 (**A**), S3 (**C**), and S5 (**E**), and those of ochratoxin B obtained from S2 (**B**), S3 (**D**), and S5 (**F**).

The identification of an Aspergillus strains isolated from licorice

Several isolates derived from licorice root harvested in Tumxuk, Xinjiang, China, were identified using molecular and morphological methods. Spores of each isolate from potato dextrose agar (Eiken, Tokyo, Japan) slant cultures were inoculated into a microtube containing 1 mL potato dextrose broth (PDB; BD, Franklin Lakes, NJ, USA) and incubated at 25 °C for 3 days. Genomic DNA was extracted from the pellets using the sodium dodecyl sulfate method with minor modifications [37]. A partial fragment of the -tubulin gene was amplified using the Bt2a/Bt2b primer pair [38]. Amplification reactions were performed with ExTaq (TaKaRa Bio Inc., Otsu, Japan) in a thermal cycler (C1000 Touch; Bio-Rad Laboratories, Inc., Hercules, CA, USA) according to the manufacturer's instructions. The amplified DNA strands were sequenced using the BigDye Terminator cycle sequencing kit (version 3.1; Life Technologies, Carlsbad, CA, USA) and ABI 3730xl genetic analyzer (Life Technologies). The obtained sequences were phylogenetically analyzed against reference sequences from the *Aspergillus* section *Circumdati* referred from Frisvad et al. [39] using the neighbor-joining method [40] and MEGA X [38]. Based on the phylogenetic relationships, the isolates were identified as *A. westerdijkiae*. They were inoculated onto Czapek yeast extract agar [containing 35 g/L Czapek-Dox broth (BD), 5 g/L yeast extract, and 20 g/L agar], and malt extract agar (Oxoid, Basingstoke, UK), and incubated at 25 °C for 7 days. After incubation, the isolates were confirmed to be *A. westerdijkiae* with reference to Frisvad et al. [39] based on their macroscopic and microscopic features.
